# Supplementary material for: Choice of respiratory therapy for COVID-19 patients with acute hypoxemic respiratory failure: a retrospective case series study
Source: PeerJ. 2023 Apr 10;11:e15174. doi: 10.7717/peerj.15174 (PMC10100803; doi:10.7717/peerj.15174)
Supplement: Supplemental Information 1 [file peerj-11-15174-s001.pdf]

Table S1. Complications of HFNC and MV groups

characteristics	HFNC	MV	*p*-value
Complications			
smoking history, n (%)	15 (43%)	14 (58%)	0.286
hypertension, n (%)	14 (40%)	12 (50%)	0.447
diabetes, n (%)	18 (51%)	12 (50%)	0.914
chronic kidney disease, n (%)	7 (20%)	12 (50%)	0.015*
chronic heart failure, n (%)	4 (11%)	2 (8.3%)	1.000
chronic lung disease n (%)	2 (6%)	2 (8.3%)	1.000
asthma, n (%)	2 (6%)	1 (4.2%)	1.000
stroke, n (%)	0 (0%)	2 (8.3%)	0.407

*$p<0.05$, statistically significant difference between HFNC and MV. HFNC, high flow nasal cannula; MV, mechanical ventilation.
