# Supplementary material for: Choice of respiratory therapy for COVID-19 patients with acute hypoxemic respiratory failure: a retrospective case series study
Source: PeerJ. 2023 Apr 10;11:e15174. doi: 10.7717/peerj.15174 (PMC10100803; doi:10.7717/peerj.15174)
Supplement: Supplemental Information 2 [file peerj-11-15174-s002.pdf]

Table S2. Therapeutic interventions of HFNC and MV groups

Interventions	HFNC, n(%)	MV,n(%)	*p*-value
Favipiravir			
Favipiravir	0 (0%)	1 (4.2%)	0.407
Favipiravir+Heparin+Dexamethasone	2 (5.7%)	2 (8.3%)	1.000
Favipiravir+Heparin+Dexamethasone+Steroid pulse	1 (2.9%)	0 (0%)	1.000
Remdesivir			
Remdesivir+Heparin+Dexamethasone	10 (28.6%)	3 (12.5%)	0.143
Remdesivir+Heparin+Dexamethasone+Baricitinib	7 (20.0%)	7 (29.2%)	0.416
Remdesivir+Heparin+Dexamethasone+Steroid pulse	1 (2.9%)	0 (0%)	1.000
Remdesivir+Heparin+Dexamethasone+Tocilizumab	5 (14.3%)	7 (29.2%)	0.198
Remdesivir+Heparin+Dexamethasone+Steroid pulse+Baricitinib	1 (2.9%)	0 (0%)	1.000
Remdesivir+Heparin+Steroid pulse	0 (0%)	1 (4.2%)	0.407
Favipiravir+Remdesivir			
Favipiravir+Remdesivir+Heparin+Dexamethasone	6 (17.1%)	3 (12.5%)	0.725
Favipiravir+Remdesivir+Heparin +Dexamethasone+Tcilizumab	1 (2.9%)	0 (0%)	1.000
Favipiravir+Remdesivir+Heparin +Dexamethasone+Baricitinib	1 (2.9%)	0 (0%)	1.000

HFNC, high flow nasal cannula; MV, mechanical ventilation.
