# Supplementary material for: Choice of respiratory therapy for COVID-19 patients with acute hypoxemic respiratory failure: a retrospective case series study
Source: PeerJ. 2023 Apr 10;11:e15174. doi: 10.7717/peerj.15174 (PMC10100803; doi:10.7717/peerj.15174)
Supplement: Supplemental Information 3 [file peerj-11-15174-s003.pdf]

Table. S3. Clinical outcomes of HFNC and MV groups after admission

characteristics	HFNC	MV	*p*-value
hospital stay, days, mean ± sd (range)	13.2 ± 10.3 (4-63)	24.1 ± 21.2 (5-97)	0.032*
Period from onset to the introduction of ventilator management, days, mean ± sd (range)		10.3 ± 4.3 (4-22)	
Period from HFNC to mechanical ventilation, days, mean ± sd (range)		2.8 ± 3.6 (0-16)	
Period of using HFNC, days, mean ± sd (range)	7.1 ± 10.3 (1-62)		
Period of using ventilation, days, mean ± sd (range)		15.2 ± 23.6 (2-97)	

*$p<0.05$, statistically significant difference between HFNC and MV. HFNC, high flow nasal cannula; MV, mechanical ventilation.
