# Supplementary material for: Choice of respiratory therapy for COVID-19 patients with acute hypoxemic respiratory failure: a retrospective case series study
Source: PeerJ. 2023 Apr 10;11:e15174. doi: 10.7717/peerj.15174 (PMC10100803; doi:10.7717/peerj.15174)
Supplement: Supplemental Information 4 [file peerj-11-15174-s004.pdf]

Table S4 Classification by ROX index and/or LIV, and HFNC/MV ratios and mortality

Category	Mild cases, n/subtotal (%)			Severe cases, n/subtotal (%)			Total, n/subtotal (%)
	ROX index >6.1	LIV ≥35.5%	LIV< 4.26×(ROX)+7.89	ROX index ≤6.1	LIV ≥35.5%	LIV≥ 4.26×(ROX)+7.89	
HFNC	28/35 (80.0%)	31/35 (88.6%)	32/35(91.4%)	7/35 (20.0%)	4/35 (11.4%)	3/35 (8.6%)	35/59 (59.3%)
MV	6/24 (25.0%)	6/24 (25.0%)	5/24 (20.8%)	18/24 (75.0%)	18/24 (75.0%)	19/24 (79.2%)	24/59 (40.7%)
Total	34/59 (57.6%)	37/59 (62.7%)	37/59 (62.7%)	25/59 (42.4%)	22/59 (37.3%)	22/59 (37.3%)	59/59 (100%)
Mortality	2/34 (5.9%)	1/37 (2.7%)	1/37 (2.7%)	2/25 (8.0%)	3/22 (13.6%)	3/22 (13.6%)	4/59 (6.8%)

HFNC, high-flow nasal cannula; LIV, lung infiltration volume; MV, mechanical ventilation; ROX index, ratio of oxygen saturation index.
