# Supplementary material for: Choice of respiratory therapy for COVID-19 patients with acute hypoxemic respiratory failure: a retrospective case series study
Source: PeerJ. 2023 Apr 10;11:e15174. doi: 10.7717/peerj.15174 (PMC10100803; doi:10.7717/peerj.15174)
Supplement: Supplemental Information 5 [file peerj-11-15174-s005.pdf]

Table S5 Classification by ROX index and/or LIV, and the positive ratios of HFNC or MV

Category	Mild cases			Severe cases		
	HFNC positive, n/subtotal (%)			MV positive, n/subtotal (%)		
	ROX index >6.1	35.5%>LIV	LIV<4.26×(ROX)+7.89	ROX index ≤6.1	35.5%≥LIV	LIV≥4.26×(ROX)+7.89
Male	21/28 (75.0%)	22/31 (71.0%)	23/32 (71.9%)	13/18 (72.2%)	13/18 (72.2%)	15/19 (78.9%)
Female	7/28 (25.0%)	9/31 (29.0%)	9/32 (28.1%)	5/18 (27.8%)	5/18 (27.8%)	4/19 (21.1%)
Total	28/28 (100%)	31/31 (100%)	32/32 (100%)	18/18 (100%)	18/18 (100%)	21/21 (100%)
≥65 yo	9/28 (32.1%)	12/31 (38.7%)	11/32 (34.4%)	6/18 (33.3%)	8/18 (44.4%)	7/19 (36.8%)
<65 yo	19/28 (67.9%)	19/31 (61.3%)	21/32 (65.6%)	12/18 (66.7%)	10/18 (55.6%)	12/19 (63.2%)
Total	28/28 (100%)	31/31 (100%)	31/31 (100%)	18/18 (100%)	18/18 (100%)	19/19 (100%)
BMI >25	11/28 (39.3%)	9/31 (29.0%)*	11/32 (34.4%)	10/18 (55.6%)	11/18 (61.1%)	10/19 (52.6%)
BMI ≤25	17/28 (60.7%)	22/31 (71.0%)*	21/32 (65.6%)	8/18 (44.4%)	7/18 (38.9%)	9/19 (47.4%)
Total	28/28 (100%)	31/31 (100%)	32/32 (100%)	18/18 (100%)	18/18 (100%)	19/19 (100%)

BMI, body mass index; HFNC, high-flow nasal cannula; LIV, lung infiltration volume; MV, mechanical ventilation; ROX index, ratio of oxygen saturation index; yo, years old. *p<0.05 compared with 35.5%≥LIV , MV positive with χ^2-test
