# Supplementary material for: Choice of respiratory therapy for COVID-19 patients with acute hypoxemic respiratory failure: a retrospective case series study
Source: PeerJ. 2023 Apr 10;11:e15174. doi: 10.7717/peerj.15174 (PMC10100803; doi:10.7717/peerj.15174)
Supplement: Supplemental Information 6 — (A) Daily new COVID-19 cases in Kyoto Pref. (B) Total COVID-19 cases in Kyoto Pref. (C) Daily COVID-19 death in Kyoto Pref. (D) Total COVID-19 death in Kyoto Pref. (E) COVID-19 patients in this study. HFNC, High flow nasal cannula; MV, mechanical ventilation; ROX index, the ratio of oxygen saturation index. [file peerj-11-15174-s006.pdf]

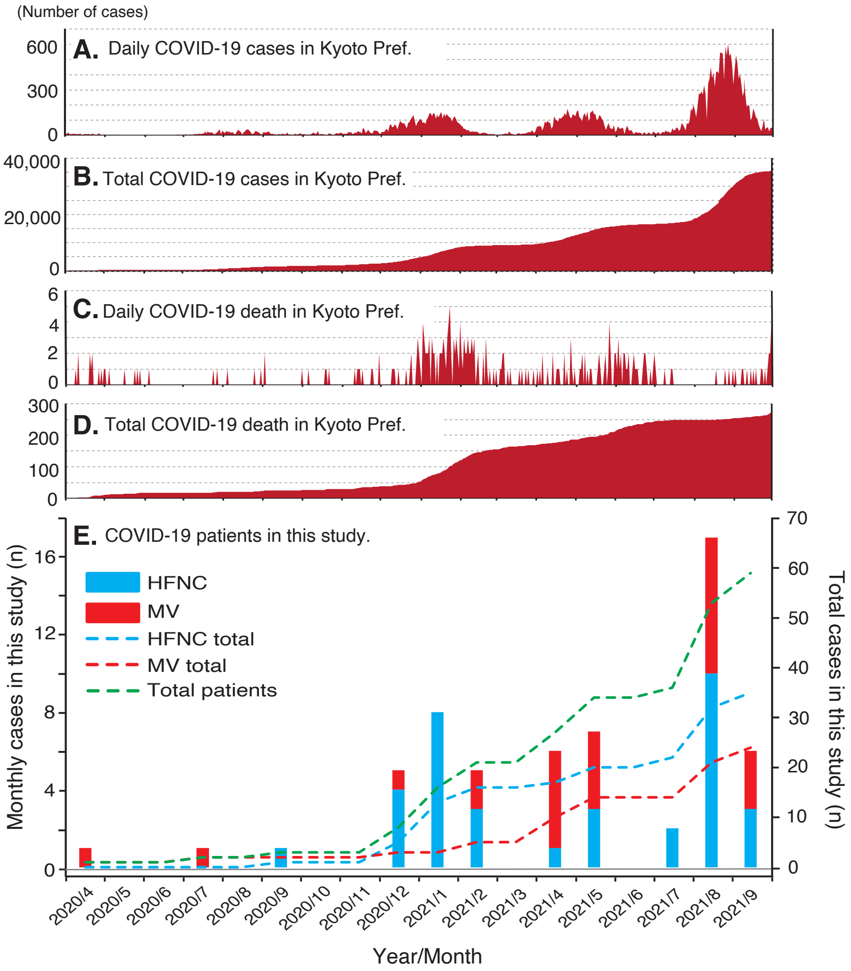

Fig. S1. The statistics of COVID-19 cases in Kyoto Prefecture and monthly COVID-19 patients in this study. **A.** Daily new COVID-19 cases in Kyoto Pref. **B.** Total COVID-19 cases in Kyoto Pref. **C.** Daily COVID-19 death in Kyoto Pref. **D.** Total COVID-19 death in Kyoto Pref. **E.** COVID-19 patients in this study. HFNC, High flow nasal cannula; MV, mechanical ventilation; ROX index, the ratio of oxygen saturation index.statistics of COVID-19 patients

in Kyoto Prefecture.
