# Supplementary material for: Choice of respiratory therapy for COVID-19 patients with acute hypoxemic respiratory failure: a retrospective case series study
Source: PeerJ. 2023 Apr 10;11:e15174. doi: 10.7717/peerj.15174 (PMC10100803; doi:10.7717/peerj.15174)
Supplement: Supplemental Information 7 — BMI, body mass index; CCI, Charlson comorbidity index; Corr, correlation coefficient; CRP, C-reactive protein (mg/L); Cr, serum creatinine (mg/dL); DtoH, Period from onset to admission to our hospital (days); DtoHF, Period from onset to the introduction of HFNC (days); Ddim, d-dimer (mg/L); HFNC, gender (red: male, blue: female); High flow nasal cannula; LIV, lung infiltration volume (%); LDH, lactate dehydrogenase (U/L); MV, mechanical ventilation; PSI, Pneumonia severity index; ROX index, the ratio of oxygen saturation index; WBC, white blood cells (/ μL) [file peerj-11-15174-s007.pdf]

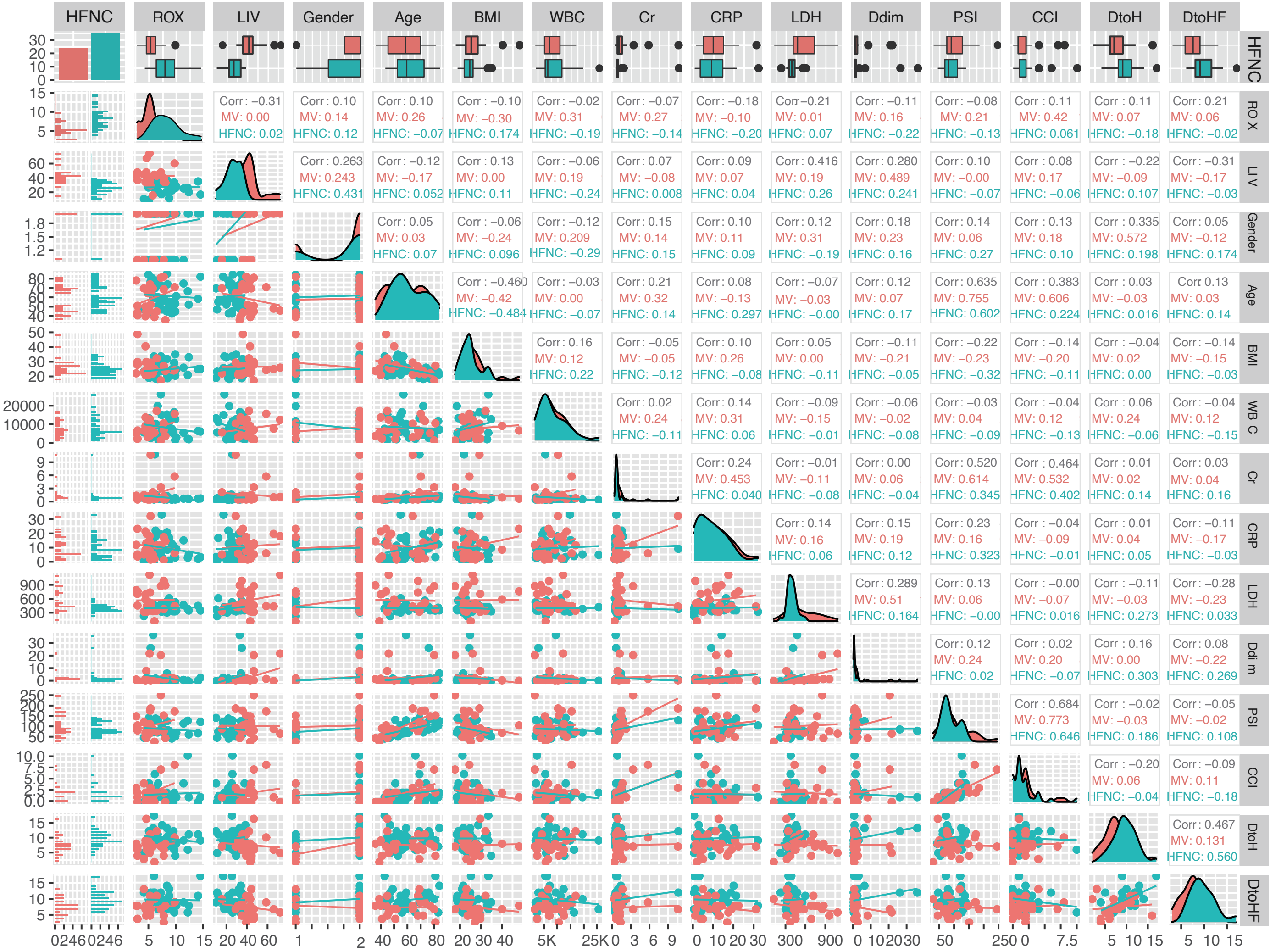


**Fig. S2.** Pairs plot showing the difference in the distribution of each analysis factor when divided into two groups, the HFNC group (blue-colored) and the MV group (red-colored), using ggpairs() of GGally packages of R programming language. BMI, body mass index; CCI, Charlson comorbidity index; Corr, correlation coefficient; CRP, C-reactive protein (mg/L); Cr, serum creatinine (mg/dL); DtoH, Period from onset to admission to our hospital (days); DtoHF, Period from onset to the introduction of HFNC (days); Ddim, d-dimer (mg/L); HFNC, gender (red: male, blue: female); High flow nasal cannula; LIV, lung infiltration volume (%); LDH, lactate dehydrogenase (U/L); MV, mechanical ventilation; PSI, Pneumonia severity index; ROX index, the ratio of oxygen saturation index; WBC, white blood cells (/µL)
