# Supplementary material for: Choice of respiratory therapy for COVID-19 patients with acute hypoxemic respiratory failure: a retrospective case series study
Source: PeerJ. 2023 Apr 10;11:e15174. doi: 10.7717/peerj.15174 (PMC10100803; doi:10.7717/peerj.15174)
Supplement: Supplemental Information 8 — (A). gender, (B). age (≧ 65, 65 >), (C). BMI (≧ 25, 25 >). The borderline to classify patients with HFNC and ventilatory management was (LIV) = 4.51 ×(ROX index) +1.75. ROX index and LIV were significant in multiple logistic regression analyses. BMI, body mass index; HFNC, High flow nasal cannula; LIV, lung infiltration volume; MV, mechanical ventilation; ROX index, the ratio of oxygen saturation index. [file peerj-11-15174-s008.pdf]

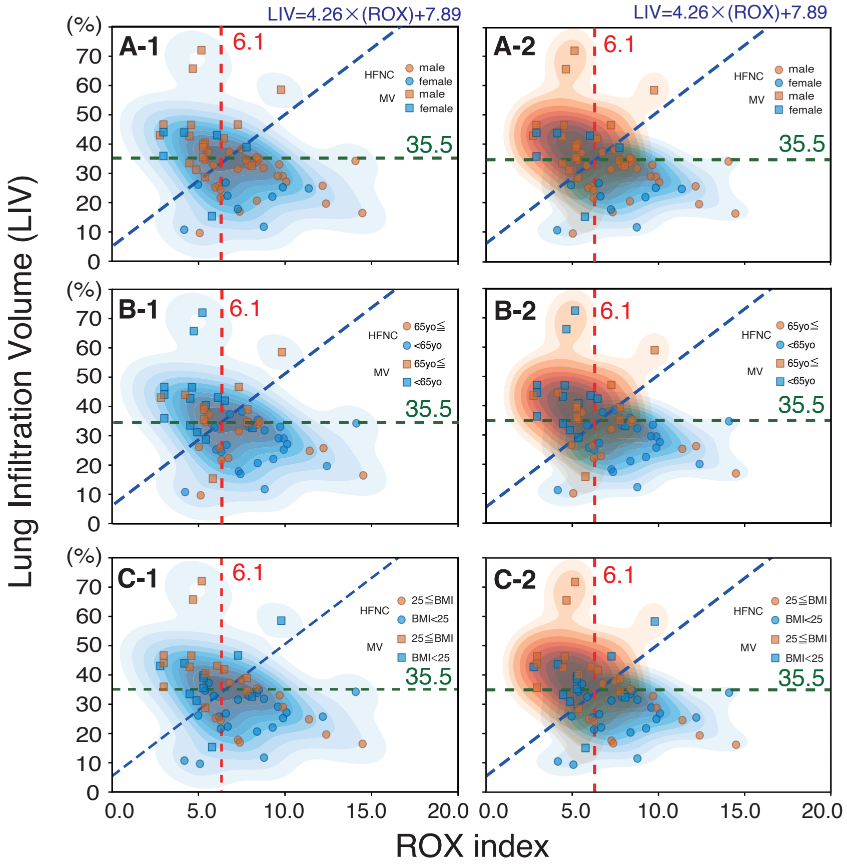

Supplementary Fig. 3. Kernel density estimation of patient distribution

A; gender, B; age (≧65, 65>), C; BMI (≧25, 25>). The borderline to classify patients with HFNC and ventilatory management was (LIV)= 4.51×(ROX index) +1.75. ROX index and LIV were significant in multiple logistic regression analyses.

BMI, body mass index; HFNC, High flow nasal cannula; LIV, lung infiltration volume; MV, mechanical ventilation; ROX index, the ratio of oxygen saturation index.
